# Study on the Effect of Salt Stress on Yield and Grain Quality Among Different Rice Varieties

**DOI:** 10.3389/fpls.2022.918460

**Published:** 2022-05-31

**Authors:** Rui Zhang, Yang Wang, Shahid Hussain, Shuo Yang, Rongkai Li, Shuli Liu, Yinglong Chen, Huanhe Wei, Qigen Dai, Hongyan Hou

**Affiliations:** ^1^Jiangsu Key Laboratory of Crop Genetics and Physiology, Jiangsu Key Laboratory of Crop Cultivation and Physiology, Jiangsu Co-Innovation Center for Modern Production Technology of Grain Crops, Research Institute of Rice Industrial Engineering Technology, Key Laboratory of Saline-Alkali Soil Improvement and Utilization (Coastal Saline-Alkali Lands), Ministry of Agriculture and Rural Affairs, Yangzhou University, Yangzhou, China; ^2^Yibang Agriculture Technology Development Co., Ltd., Dongying, China

**Keywords:** rice (*Oryza sativa* L.), salt stress, yield, grain quality, mineral elements

## Abstract

Salt is one of the main factors limiting the use of mudflats. In this study, the yield, quality, and mineral content of rice seeds under salt stress were investigated. A pot experiment was conducted with Yangyugeng2, Xudao9, and Huageng5 under 0, 17.1, 25.6, and 34.2 mM NaCl of salt concentration treatments. The results showed that salt stress can significantly decrease panicle number, grain number per panicle, 1000-grain weight and yield of rice, and the panicle number was among other things the main cause of yield loss under saline conditions. When the salt concentration is less than 34.2 mM NaCl, the salt stress increases the brown rice rate and milled rice rate, thus significant increasing head milled rice rate of salt-sensitive varieties but decreasing in salt-tolerant varieties. In addition, the grain length is more sensitive than grain width to salt stress. This study also indicates that different varieties of rice exhibit different salt tolerance under salt stress, the three rice varieties in this study, in order of salt tolerance, are Xudao9, Huageng5, and Yangyugeng2. Salt stress will increase the appearance, viscosity, degree of balance, and taste value, and decrease the hardness of rice when salt concentration is less than 17.1 mM NaCl in Yangyugeng2 and Huageng5 or 25.6 mM NaCl in Xudao9. The differences in starch pasting properties among rice varieties in this study are larger than those caused by salt stress. The uptake capacity of K, Mg, P, S, and Cu ions in the seeds of different rice varieties significantly vary, and salt stress causes significant differences in the uptake capacity of K, Na, and Cu ions in rice seeds. Rice varieties with high salt tolerance can be selected for the development and utilization of mudflats, and low concentration of salt stress will increase the rice quality, all of which are meaningful to agricultural production.

## Introduction

Rice (*Oryza sativa* L.) is one of the major food sources; with the increase of population worldwide, the demand for rice will grow faster than for other crops (Krishnan et al., 2011). However, the global rice yield is deeply affected by salinity stress (Corwin, 2020; Ltaeif et al., 2021; Sangwongchai et al., 2021).

Soil salinity is one of the most common environmental stressors; about 20% of arable land and nearly half of irrigated land are being affected by salt stress in the world (Fahad et al., 2019; Dramalis et al., 2021). Saline soils are unevenly distributed throughout the world, with Oceania, Asia, America, Africa, and Europe accounting for 37.42, 33.43, 15.39, 8.43, and 5.32%, respectively (Jianfeng, 2008). In Asia, China has the largest area of saline soils. Soil salinization causes soil nutrient loss, destroys the structure of soil aggregates, and significantly affects crop growth and development (Kordrostami et al., 2017). Jiangsu Province of China has the largest coastal tidal flat that is not only an important wetland ecosystem but also one of the lands essential to agricultural production and urban development in coastal areas (Hua et al., 2017).

Under saline stress, plants have difficulty forming both firmness and hardness in the soil required for effective root systems. The low water availability causes osmotic stress and reduces efficiency in transporting nutrients such as nitrogen, phosphorus, potassium, and calcium, thereby causing nutrient deficiencies and ionic toxicity (Razzaq et al., 2020). When the electrical conductivity of irrigation water is greater than 32.8 mM NaCl, rice yield will be reduced (Eynard et al., 2005; Chen et al., 2020).

Saline soil affects both growth and yield of rice plants, and the nutrient content of rice, resulting in an imbalance of ion that affects the quality of the rice (Rao et al., 2013). Rice quality, a multi-faceted trait (Fitzgerald et al., 2009), is the result of interactions among rice genes, surrounding environment, cultivation management methods, milling conditions, and storage technologies (Han et al., 2004). The yield and quality of rice determine consumer purchasing behaviors and the ruling prices in the market (Balindong et al., 2018; Sangwongchai et al., 2021). Consumers mainly evaluate the quality of rice by its physical (milled yield, transparency, size, shape, and color) and organoleptic (cooking characteristics) properties (Fahad et al., 2019). Rice quality includes the quality of milling, appearance, cooking and eating, and nutrition (Bian et al., 2018). The appearance quality includes the shape and chalkiness of fine rice. The cooking and eating quality includes appearance, hardness, viscosity, and degree of balance; the nutritive quality includes amylose content, gel consistency, the protein content of rice (Fitzgerald et al., 2009). These characteristics are the main factors that influence the market price of rice.

Mineral elements play an important role in human health and are the main components of food (Wang et al., 2017; Razzaq et al., 2020). What people mainly consume is refined rice that is low in nutrients such as minerals and vitamins, which creates hidden hunger (micronutrient deficiencies) in developing countries where rice is a staple food (Fitzgerald et al., 2009). Micronutrient deficiencies such as copper and zinc may cause harmful effect such as hypoimmunity. Calcium is needed for proper bone growth and development, and manganese is associated with many enzymes in the body. Manganese deficiency may lead to venous sclerosis (Jiang et al., 2008).

Most of the previous literature (Menezes-Benavente et al., 2004; Nounjan et al., 2012; Senguttuvel et al., 2013) studied the effect of salt stress on the enzyme activity and gene expression of rice, but only a few researched the effect of salt stress on rice grain quality. A lot of literature (Limin, 2012; Mei et al., 2015; Thu et al., 2017) has studied the effect of salt stress on mineral element content of various organs of rice plants, but only a few studied on the mineral element content of rice seeds. In the present study, we selected Yangyugeng2, Xudao9, and Huageng5, which have been widely growing in Jiangsu Province, as the test objects. We aim to understand the effect of different salinity stress on rice grain quality and mineral element content of rice seeds.

## Materials and Methods

### Experiment Materials and Experimental Designs

A pot experiment was carried out under rain shelters in the experimental field of Agricultural College of Yangzhou University, Yangzhou City, Jiangsu Province, China (119°25′E, 32°30′N) from May to November in 2019. The two-factor experiments were laid out in a completely randomized design with ten replicates, where the primary factor was salt concentration, and the secondary factor was rice varieties. The threshold value indicates the maximum salinity allowed without reducing the yield, and the threshold of rice is 32.8 mM NaCl (Das et al., 2015). The four salinity levels (0, 17.1, 25.6, and 34.2 mM NaCl) were set for this experimental study. The rice varieties used in this study were Yangyugeng2, Xudao9, and Huageng5, and these three cultivars have been widely grown in Jiangsu Province. The seeds with full-grain and the same size were selected and sterilized with prochloraz and aldimorph cartap for 3 days and rinsed with pure water three times to wash away the residues. Cleaned seeds were sown on 23 May, and the 28-day-old rice seedlings were transplanted to pots that were 27 cm high with the top and bottom diameters of 30 and 22 cm, respectively. Each pot was filled with about 15 kg of soil and planted with 4 hills, each of which was planted with four seedlings, and Each treatment has ten replicates. Before transplanting, basic fertilizers and sea salt (the main component of sea salt is sodium chloride; the ingredients are presented in Table 1 and made by Zhejiang Blue Starfish Salt Product Co., Ltd.) were mixed with soil using a potting soil mixer.

**TABLE 1:** Q/ZLY003 type instant sea crystal produced by Zhejiang Blue Starfish Salt Products Co. Ltd. test results for each ion.

Major cation	Major cation/chlorinity ratios
Potassium ion	0.016
Magnesium ion	0.069
Sodium ion	0.570
Calcium ion	0.018
Sulfate ion	0.140

Urea, superphosphate and potassium chloride were used as sources of nitrogen, phosphorus, and potassium, respectively. Urea (1.305 g/pot) was applied during the four growth stages, such as the pre-transplanting stage, tilling stage, panicle initiation stage and booting stage, respectively (Zhang et al., 2020). About 8.3 g/pot of superphosphate was applied as the basic fertilizer, and 1.665 g/pot potassium chloride was applied as the basic and panicle fertilizers.

### Determination of Rice Yield and Yield Components

Grains per panicle is the total number of grains recorded per panicle; seed setting rate is calculated as filled grain number/total grain number (Xiong et al., 2017); the 1000-grain weight was determined by randomly selecting 200 seeds and converted to the weight of 1000 seeds (Sakhare et al., 2015); rice seeds were hand-threshed, with the moisture content being adjusted to 14%, and the total seed weight per pot recorded as rice yield.

### Determination of Milling Quality

A total of 500 g of grain harvested from each treatment was dried in sunshine till the grain moisture of 14% was obtained for quality analysis (Zhu et al., 2017). A 150 g rice sample passed through the rice huller twice (SY88-TH, South Korea) to obtain brown rice that would then pass through the rice polisher (LTJM-2099, made by Zhejiang Bethlehem Apparatus Co., Ltd.) to obtain milled rice. Head milled rice constituted the grain with length of 3/4 or more of the whole milled grain separated from the 30 g milled rice (Kaur et al., 2016). The computational formula of brown rice rate, milled rice rate, and head milled rice are as follows:


$$\mathrm{brown}\,\mathrm{rice}\,\text{rate}=\frac{\mathrm{brown}\,\mathrm{rice}\,\text{weight}}{150}*100\%$$

$$\mathrm{milled}\,\mathrm{rice}\,\text{rate}=\frac{\mathrm{milled}\,\mathrm{rice}\,\text{weight}}{150}*100\%$$

$$\mathrm{head}\,\mathrm{milled}\,\mathrm{rice}\,\text{rate}=\frac{\mathrm{milled}\,\mathrm{rice}\,\text{weight}}{30}*\mathrm{milled}\,\mathrm{rice}\,\text{rate}$$

### Determination of Appearance Quality

The length, width, aspect ratio, chalkiness rate, and chalkiness degree of head milled rice were measured using a grain appearance analyzer ScanMaker i800 plus (Microtek, China) (Zhao et al., 2018).

### Determination of Nutritive Quality

N concentration in rice flour was determined based on the Kjeldahl method (Hernandez-Santana et al., 2019), and the protein content was derived by multiplying the product of nitrogen content by 5.95 (Hu et al., 2020). The amylose content was determined using iodine colorimetry (Shaik et al., 2014); The gel consistency was determined based on the methods of Cagampang et al. (1973).

### Determination of Cooking/Eating Quality

Rice taste analyzer (Satake, Hiroshima, Japan) was used to assess the taste value of rice grains. The taste value is a comprehensive evaluation of cooked rice and includes the appearance, hardness, viscosity, and degree of balance (Zhang et al., 2020). A sample containing 30 g of polished grains from each cultivar was placed into a stainless-steel tank, rinsed with running purified water for 30 s, drained, and reconstituted with purified water to bring the ratio of rice to water to 1:1.33. The sample was then soaked for 30 min in the tank, covered with a filter paper, and sealed with a rubber ring. The stainless-steel tank was placed into an electric rice cooker (JT783, Midea, Shunde, China), covered, steamed for 30 min, and kept warm for 10 min. The tank was taken out from the rice cooker, while gently stirring and turning over the steamed rice. Then, the tank was covered with a filter paper again and cooled for 20 min using a supporting air-cooling device. Afterward, the filter paper was replaced with the supporting steel cover to seal and cool the steamed rice at room temperature (25°C) for 90 min. An 8-g sample of steamed rice was placed into a stainless-steel ring (30-mm diameter and 9-mm height) and then pressed the rice into a cake. The rice cake was placed in a measuring tank, and the rice taste analyzer was inserted to measure appearance and taste value of the steamed rice sample. Three rice cakes were measured for each steamed rice sample, and the top and bottom surface of each cake was measured once (Zhu et al., 2021b).

### Determination of Pasting Properties of Starch

The pasting properties of rice flour were determined by rapid viscosity analyzer (RVA-TecMaster, Perten, Stockholm, Sweden), according to Zhu et al. (2017). Rice flour (3 g, which moisture content of the rice flour totaled 12%; if the moisture content of rice flour was not 12%, it is necessary to increase or decrease the weight of rice flour appropriately) was weighed and placed into an RVA sample canister and 25 mL of distilled water was added; then the sample canister was transferred into the RVA for testing. The entire program cycle was 13 min. Starch samples were cycled through a heating-cooling program starting at 50°C for 1 min, then raising the temperature from 50 to 95°C at a rate of 12°C/min, holding at 95°C for 2.5 min, cooling to 50°C at 12°C/min, and ending at another hold at 50°C for 2 min. The pasting parameters were recorded for analysis (Zhu et al., 2021b). The RVA profile characteristics include such common parameters as peak viscosity, hot paste viscosity, cold paste viscosity, breakdown of secondary parameters, setback, peak time, and pasting temperature (Zhu et al., 2021a). The parameters were calculated according to the formula below (Bao, 2019):


breakdown=peak⁢viscosity-hot⁢paste⁢viscosity

setback=cold⁢paste⁢viscosity-peak⁢viscosity

### Determination of Mineral Element Contents

The milled rice of Yangyugeng2, Xudao9, and Huageng5 was ground to powder and sieved through a 100-mesh sieve. A total of 0.45 g dried samples was weighed, and 5 mL HNO3 (GR) and 3 mL ultrapure water, together with 2–3 drops of hydrogen peroxide, were added; then the samples were digested in a microwave digester (MARS 5, CEM Corporation, NC, United States). After digestion, the volume was fixed at 50 mL, filtered through a double-layer quantitative filter paper. The filtered liquid were used to measure the Cu element and ten-times dilution filtrate, which was used for testing macro element, was determined by ICP-AES (IRIS Intrepid II XSP, Thermo Scientific, MA, United States) (Dongfen et al., 2009; Domingo et al., 2016; Qing et al., 2019). The mineral elements were counted according to the following formula:


$$\mathrm{concentration}\,\mathrm{of}\,\mathrm{macro}\,\text{elements}=\frac{\mathrm{tested}\,\text{value}*10*50}{1000*\text{weight}}\,\text{mg}/\text{g}$$

$$\mathrm{concentration}\,\mathrm{of}\,\mathrm{Cu}\,\text{elements}=\frac{\mathrm{tested}\,\text{value}*50}{\text{weight}}\,\text{mg}/\text{kg}$$

### Statistical Analysis

Data were analyzed by a Two-way ANOVA to assess the effect of salinity stress on the rice yield, yield component, milling quality of rice grain, appearance quality, nutritive quality, cooking/eating quality, pasting properties of starch and mineral elemental contents of rice seeds. When the treatment means were different, the Duncan multiple range test was performed to identify significant differences at *P* < 0.05. Data arrangement and sorting were processed by Microsoft Excel 2016 and analyzed using IBM SPSS Statistics 26 software (IBM, Armonk, NY, United States), thus generating graphs using Origin 2018 (Origin Lab, Hampton, MA, United States).

## Results

### Effect of Salt Stress on Yield and Yield Components of Rice

**FIGURE 1:**
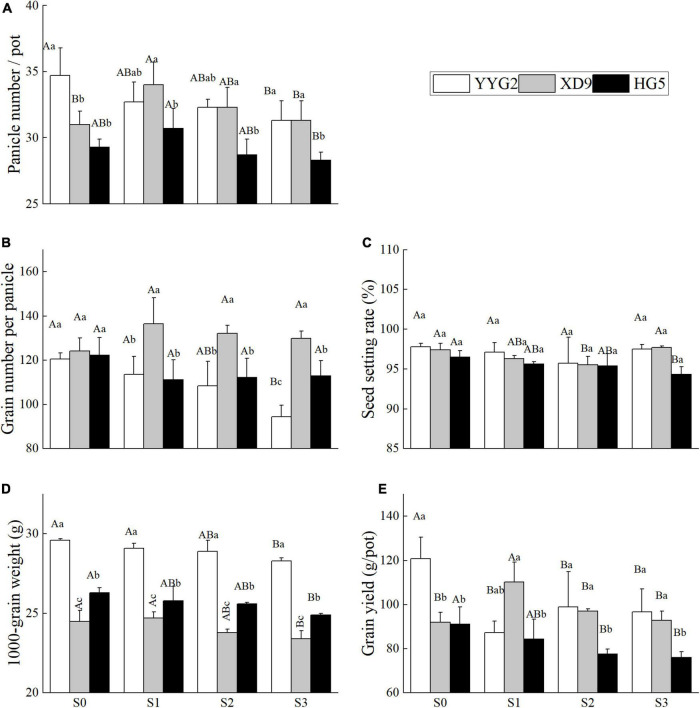
Effect of salt stress on panicle number **(A)**, grain number per panicle **(B)**, seed setting rate **(C)**, 1000-grain weight **(D)**, and grain yield **(E)** for different rice varieties. YYG2, Yangyugeng2; XD9, Xudao9; HG5, Huageng5; V, Variety; S, Salt; S_0_, S_1_, S_2_, and S_3_ denote salt concentrations of 0, 17.1, 25.6 and 34.2 mM, respectively; In different salinity level during the same varieties, bars with the same uppercase letters are not significantly different, while within the same salt concentration in different varieties, bars with the same lowercase letters are not significantly different according to the Duncan test at *P* < 0.05.

As indicated in Figure 1, salt stress had significant effect on the panicle number (*P* = 0.001), grain number per panicle (*P* = 0.001), seed setting rate (*P* = 0.010), 1000-grain weight (*P* < 0.001) and rice yield (*P* < 0.001), and it was noticeable that significant differences were also detected among rice cultivars (*P* < 0.050). There was significant interaction effect between salt treatments and rice varieties for 1000-grain weight (*P* < 0.001), but no significant interaction effect for panicle number (*P* = 0.947), grain number per panicle (*P* = 0.309), seed setting rate (*P* = 0.995), and rice yield (*P* = 0.373). The S3 treatments significantly decreased by 9.8% in the panicle number, 21.6% in the grain number per panicle, 4.4% in the 1000-grain weight, and 20% in the rice yield compared with the control (without salt treatment) in Yangyugeng2. The 1000-grain weight, rice yield and grain number per panicle of Yangyugeng2 and Huageng5 were reduced with the increasing concentration of salt by 0.8, 19.9, and 9.9% at S1 treatment and further declined as the salt concentration increased in Xudao9. The seed setting rate of Huageng5 decreased with the increasing level of salinity, while that of Yangyugeng2 and Xudao9 reduced as the concentration of salt increased by 1.88 and 2.3% at S3 treatment when the salinity level was less or equal to S2 treatment.

From the effect of salt concentration stress on yield composition, it can be seen that salt stress significantly reduced the panicle number, 1000-grain weight and yield of rice, and low concentration of salt stress increased the panicle number of Xudao9 and Huageng5, where Xudao9 reached a significant level, but Huageng5 did not. While the same salt concentration will reduce the number of spikes of Yangyugeng2 well, which indicates that the tolerance of Xudao9 to salt stress is greater than that of Huageng5 and Yangyugeng2. From the effect of salt concentration stress on the 1000-grain weight of rice seeds, the difference between rice varieties was greater than that of salt stress effect. From the effect of salt concentration stress on yield, the salt tolerance of Xudao9 was greater than that of Yangyugeng2 and Huageng5. The number of spike grains of Xudao9 and Huageng5 did not change significantly under salt concentration stress, while that of Yangyugeng2 showed a significant decrease.

### Effects of Salt Stress on the Quality of Rice Milling, Appearance, and Nutrition

**FIGURE 2:**
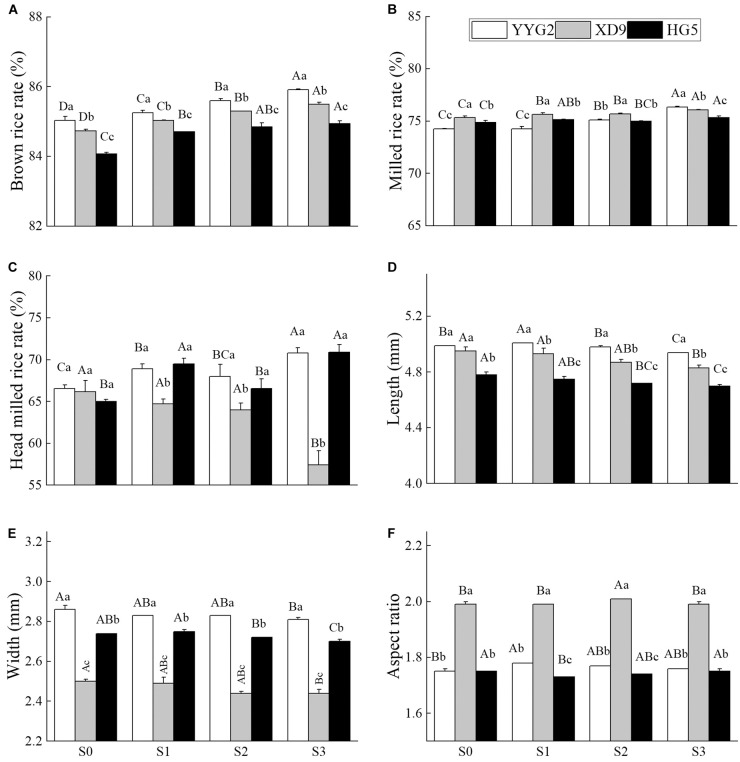
Effect of salt stress on brown rice rate **(A)**, milled rice rate **(B)**, head milled rice rate **(C)**, length **(D)**, width **(E)**, and aspect ratio **(F)** for different rice varieties. YYG2, Yangyugeng2; XD9, Xudao9; HG5, Huageng5; V, Variety; S, Salt; S_0_, S_1_, S_2_, and S_3_ denote salt concentrations of 0, 17.1, 25.6 and 34.2 mM, respectively; In different salinity level during the same varieties, bars with the same uppercase letters are not significantly different, while within the same salt concentration in different varieties, bars with the same lowercase letters are not significantly different according to the Duncan test at *P* < 0.05.

The results showed that salt stress significantly increased both brown rice rate and milled rice rate of rice, and variety differences had significant effect on the brown rice rate (*P* < 0.001), milled rice rate (*P* < 0.001), head milled rice rate (*P* < 0.001), and rice seed length (*P* < 0.001), width (*P* < 0.001) and aspect ratio (*P* < 0.001). It was noticeable that significant differences were also detected under salt stress except for the aspect ratio (*P* = 0.280). There was significant interaction effect between salt stress and rice varieties in terms of brown rice rate (*P* < 0.001), milled rice rate (*P* < 0.001), head milled rice rate (*P* < 0.001) and aspect ratio (*P* = 0.005), and no significant interaction effect on both length (*P* = 0.130) and width (*P* = 0.248) of rice. This study showed that the brown rice rate and milled rice rate of rice varieties significantly increased with the rising of salinity level, compared with the rates in terms of salinity level (Figures 2A,B). The head milled rice rate of milling quality under salt stress significantly increased in Yangyugeng2, compared with that of the control. On the contrary, the head milled rice rates of S1, S2, and S3 reduced by 2.22, 3.31, and 12.23% in Xudao9.

**FIGURE 3:**
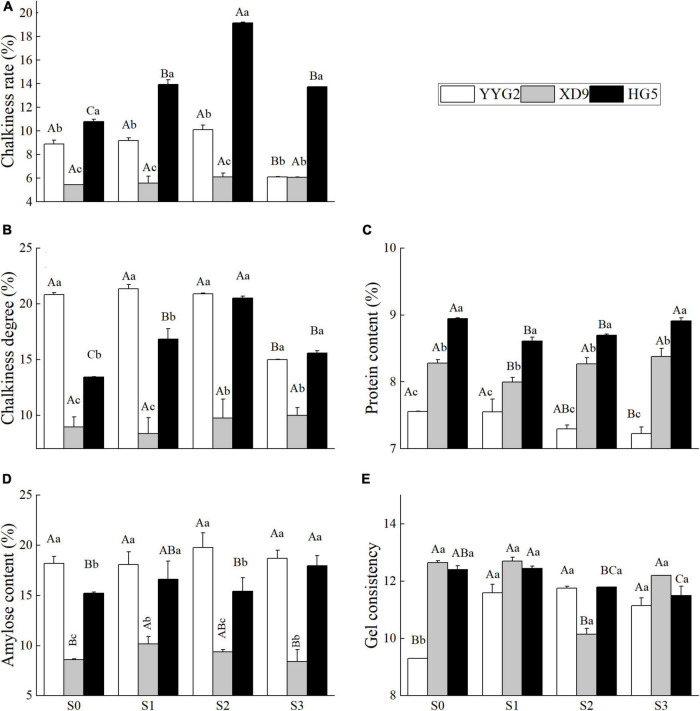
Effect of salt stress on chalkiness rate **(A)**, chalkiness degree **(B)**, protein content **(C)**, amylose content **(D)**, gel consistency **(E)** for different rice varieties. YYG2, Yangyugeng2; XD9, Xudao9; HG5, Huageng5; V, Variety; S, Salt; S_0_, S_1_, S_2_, and S_3_ denote salt concentrations of 0, 17.1, 25.6 and 34.2 mM, respectively; In different salinity level during the same varieties, bars with the same uppercase letters are not significantly different, while within the same salt concentration in different varieties, bars with the same lowercase letters are not significantly different according to the Duncan test at *P* < 0.05.

With the increase in salt concentration, the chalkiness rate and chalkiness degree showed an increasing and then decreasing trend under salt stress (Figures 3A,B), and the aspect ratio of Yangyugeng2 and Xudao9 first increased by 1.7 and 1.0% and then decreased (Figure 2F). The aspect ratio of Huageng5 first decreased by 1.7% and then increased (Figure 2F). The chalkiness rate reached its maximum in all varieties when the salt concentration was at 25.6 mM NaCl (Figure 3A). The chalkiness degree of Yangyugeng2 and Huageng5 gradually increased under salinity stress and reached the maximum value (21.36, 20.52) when the salt concentrations were 17.1 and 25.6 mM NaCl. However, the chalkiness degree of Xudao9 declined to the minimum value (8.36) at 17.1 mM NaCl and increased when the salt concentration was greater than 17.1 mM NaCl (Figure 3B).

This study indicated that significant difference among rice varieties were observed on the chalkiness rate (*P* < 0.001), chalkiness degree (*P* < 0.001), protein content (*P* < 0.001), and amylose content (*P* < 0.001), and it was noticeable that significant differences were also detected under salt stress except for the gel consistency (*P* = 0.323). There was significant interaction effect between salt treatment and rice varieties in terms of chalkiness rate (*P* < 0.001), chalkiness degree (*P* < 0.001) and protein content (*P* = 0.001), and no significant interaction effect for amylose content (*P* = 0.071) and gel consistency (*P* = 0.071) of rice seeds. The nutritive quality of rice was mainly determined by protein content, amylose content and gel consistency in rice seeds. The protein content of Yangyugeng2, Xudao9, and Huageng5 was thus reduced by 1.8, 1.8, and 9.7% when the salt concentration was 17.1 mM NaCl, which showed a tendency to increase while the salinity level was greater than 17.1 mM NaCl. However, the situation of amylose content was in the opposite (Figures 3C,D). As salt concentration increased, a trend of first rising and then falling was seen (Figure 3D). With the increase of salt concentration, the gel consistency presented an overall trend of increasing first and then decreasing (Figure 3E).

### Effect of Salt Stress on the Taste Value of Rice

**FIGURE 4:**
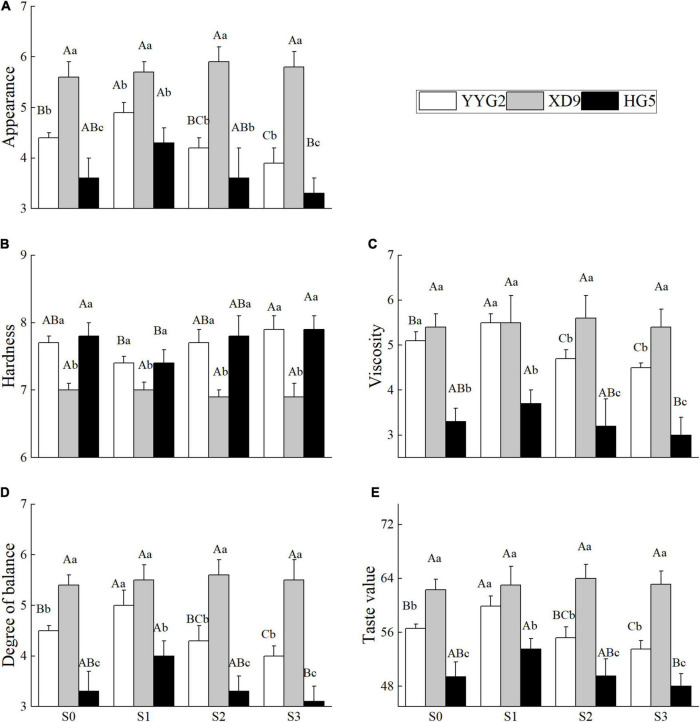
Effect of salt stress on appearance **(A)**, hardness **(B)**, viscosity **(C)**, degree of balance **(D)**, and taste value **(E)** for different rice varieties. YYG2, Yangyugeng2; XD9, Xudao9; HG5, Huageng5; V, Variety; S, Salt; S_0_, S_1_, S_2_, and S_3_ denote salt concentrations of 0, 17.1, 25.6 and 34.2 mM, respectively; In different salinity level during the same varieties, bars with the same uppercase letters are not significantly different, while within the same salt concentration in different varieties, bars with the same lowercase letters are not significantly different according to the Duncan test at *P* < 0.05.

As revealed in this study, the appearance, viscosity, degree of balance, and taste values of rice under salt stress showed a trend of increasing and then decreasing, and hardness exhibited a trend of decreasing and then increasing, and salt stress had significant effect on the appearance (*P* = 0.005), hardness (*P* = 0.012), viscosity (*p* = 0.007), degree of balance (*p* = 0.006), and taste value (*p* = 0.006). It was noticeable that significant differences in the appearance (*P* < 0.001), hardness (*P* < 0.001), viscosity (*P* < 0.001), degree of balance (*P* < 0.001), and taste value (*P* < 0.001) were also detected among rice cultivars. There was no significant interaction between salt stress and rice varieties in terms of appearance (*P* = 0.132), hardness (*P* = 0.081), viscosity (*P* = 0.251), degree of balance (*P* = 0.144), and taste value (*P* = 0.160). With the increase of salt concentration, the appearance, viscosity, degree of balance, and taste value showed a first rising and then declining trend among Yangyugeng2, Xudao9, and Huageng5. However, the three rice varieties peaked at different corresponding salt concentrations, meaning that Yangyugeng2 and Huageng5 were 17.1 mM NaCl, and Xudao9 was 25.6 mM NaCl, respectively. The result showed that the appearance, viscosity, degree of balance, and taste value increased to 4.87, 5.53, 5, and 59.87, respectively, while the salt concentration was 17.1 mM NaCl, and those of Huageng5 increased to 4.27, 3.73, 3.97, and 53.5, individually. However, the salt stress had no significant effect on the taste quality of Xudao9, whose appearance, viscosity, degree of balance, and taste value increased to the maximum value (5.9, 5.6, 5.63, and 64.03) when the salt concentration was 25.6 mM NaCl (Figure 4). In contrast, the hardness of Yangyugeng2 and Huageng5 decreased to the minimum value (7.43, 7.43) when the salt concentration was 17.1 mM NaCl. When the salinity level was 25.6 mM NaCl, the hardness of Xudao9 decreased to the minimum value (6.87). The effect of salt stress on appearance, viscosity, balance and taste value showed a trend of increasing and then decreasing, and the effect on hardness demonstrated a trend of decreasing and then increasing. However, the turning points were different among rice varieties. The turning point of Xudao9 was S2 treatment, while that of Huageng5 and Yangyugeng2 was S1 treatment, indicating that the salt tolerance of Huageng5 and Yangyugeng2 was lower than that of Xudao9.

### Effect of Salt Stress on the Pasting Properties of Starch

**FIGURE 5:**
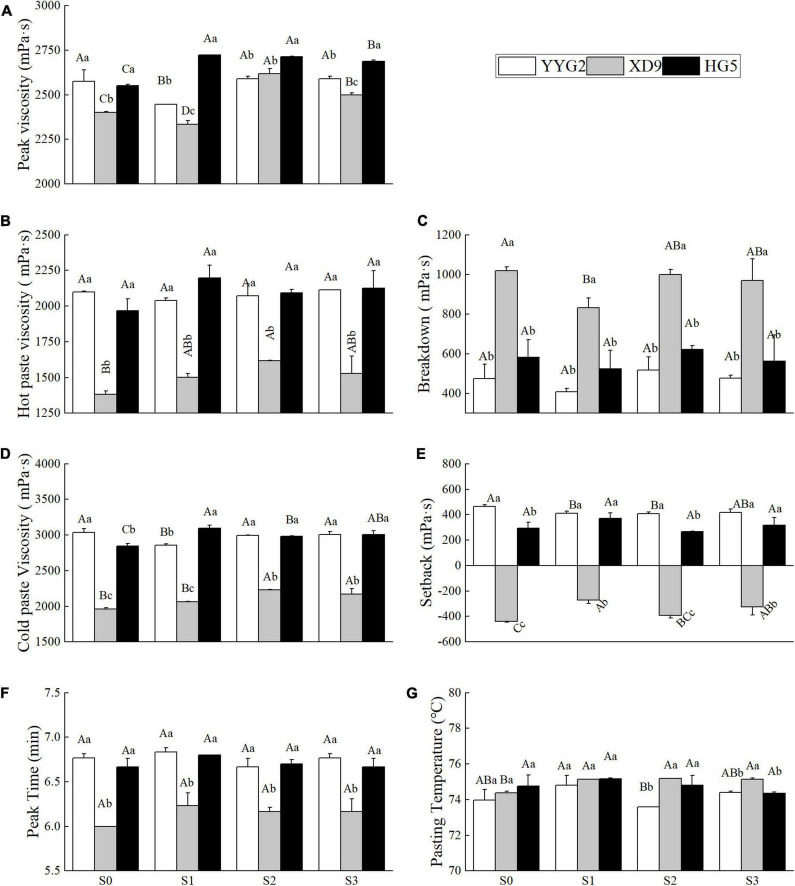
Effects of salt stress on peak viscosity **(A)**, hot paste viscosity **(B)**, breakdown **(C)**, cold paste viscosity **(D)**, setback **(E)**, peak viscosity **(F)**, and pasting temperature **(G)** for different rice varieties. YYG2, Yangyugeng2; XD9, Xudao9; HG5, Huageng5; V, Variety; S, Salt; S_0_, S_1_, S_2_, and S_3_ denote salt concentrations of 0, 17.1, 25.6 and 34.2 mM, respectively; In different salinity level during the same varieties, bars with the same uppercase letters are not significantly different, while within the same salt concentration in different varieties, bars with the same lowercase letters are not significantly different according to the Duncan test at *P* < 0.05.

The profile characteristics of RVA is a useful index to measure the cooking quality of rice. This study indicated that significant difference was observed in terms of the peak viscosity (*P* < 0.001), hot paste viscosity (*P* < 0.001), breakdown (*P* < 0.001), cold paste viscosity (*P* < 0.001), setback (*P* < 0.001), peak time (*P* < 0.001), and pasting temperature (*P* = 0.002) among rice varieties. It was noticeable that significant differences were also detected under salinity stress in terms of peak viscosity (*P* < 0.001), hot paste viscosity (*P* = 0.044), breakdown (*P* = 0.047), cold paste viscosity (*P* < 0.001), setback (*P* = 0.011), peak time (*P* = 0.048), and pasting temperature (*P* = 0.031). There was significant interaction effect between salt treatments and rice varieties in terms of peak viscosity (*P* < 0.001), cold paste viscosity (*P* < 0.001), and setback (*P* = 0.022) and no significant interaction effect for hot paste viscosity (*P* = 0.081), breakdown (*P* = 0.879), peak time (*P* = 0.414), and pasting temperature (*P* = 0.087). The experimental results revealed that the peak viscosity of Yangyugeng2, Xudao9, and Huageng5 increased to the maximum value (2589, 2618, and 2713) when the salt concentration was 25.6 mM NaCl and decreased to the minimum value (407.5, 831.5, and 525) when the salinity level was 17.1 mM NaCl, respectively (Figure 5). At the peak time, there were significant differences among rice varieties, but salinity levels were not. Furthermore, the salinity level influenced the pasting temperature when the concentration of salt was greater or equal to 25.6 mM NaCl. Salt stress increased the difference in pasting temperature among varieties, with Yangyugeng2 showing the greatest sensitivity, followed by Huageng5 and finally Xudao9 (Figure 5G).

### Effect of Salt Stress on the Mineral Element Content of Rice Seeds

**FIGURE 6:**
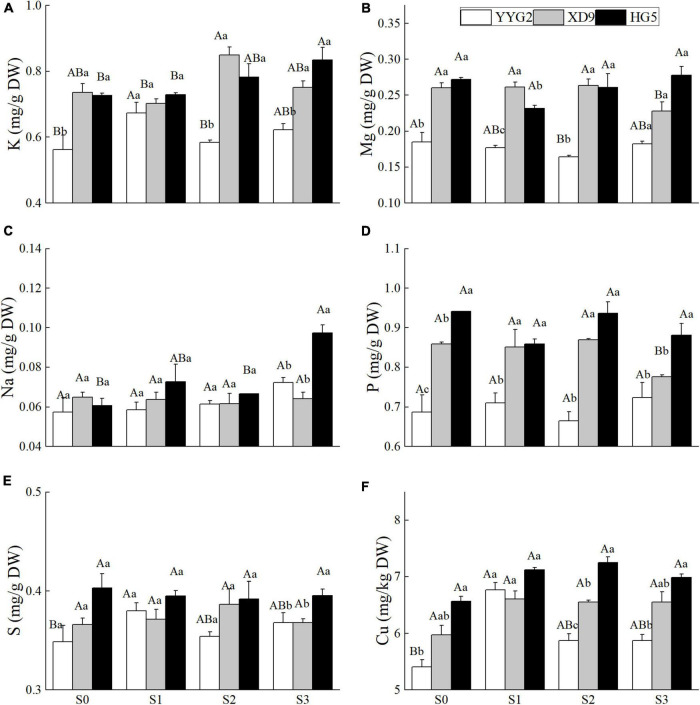
Effect of salt stress on the K **(A)**, Mg **(B)**, Na **(C)**, P **(D)**, S **(E)**, and Cu **(F)** for different rice varieties seeds. YYG2, Yangyugeng2; XD9, Xudao9; HG5, Huageng5; S_0_, S_1_, S_2_, and S_3_ denote salt concentrations of 0, 17.1, 25.6 and 34.2 mM, respectively; In different salinity level during the same varieties, bars with the same uppercase letters are not significantly different, while within the same salt concentration in different varieties, bars with the same lowercase letters are not significantly different according to the Duncan test at *P* < 0.05.

This study investigated that salt stress had significant effect on the contents of K (*P* = 0.018), Na (*P* = 0.008), and Cu (*P* = 0.003), and no significant effect on the contents of S (*P* = 0.700), P (*P* = 0.213), and Mg (*P* = 0.638). And it was noticeable that significant differences were also detected among rice cultivars except for the content of Na (*P* = 0.076). There was significant interaction effect between salt treatments and rice varieties with respect to the contents of K (*P* = 0.006), Na (*P* = 0.012), and P (*P* = 0.024), and no significant interaction effect for the contents of Mg (*P* = 0.247), S (*P* = 0.296), and Cu (*P* = 0.288). In Figure 6, the contents of K and S in the milled rice of Yangyugeng2 were the highest (0.67, 0.38) at 17.1 mM NaCl and decreased when the salt concentration was more than 17.1 mM NaCl. The contents of K and S in head milled rice of Xudao9 were the highest (0.85, 0.39) at 25.6 mM NaCl and decreased at 34.2 mM NaCl, and the contents of K and S in head milled rice of Huageng5 increased to the maximum value (0.83, 0.40) at 34.2 mM NaCl. The P content in milled rice of Xudao9 at 34.2 mM NaCl significantly decreased by 9.3% compared with that under no salinity stress. A significant difference in the P content between the rice cultivars was found. Significant differences existed among varieties in terms of the Mg content in milled rice when the salt concentration was less than or equal to 25.6 mM NaCl, and no difference was observed among cultivars at 34.2 mM NaCl. The Na content in milled rice significantly increased by 66.7% at 34.2 mM NaCl, and there were significant differences among rice cultivars. The Cu content in milled rice of Yangyugeng2 and Xudao9 increased to the maximum value (6.77, 6.61) at 17.1 mM NaCl, then declined slightly when the salinity level was greater than 17.1 mM NaCl, and that of Huageng5 increased to the maximum value (7.25) at 25.6 mM NaCl.

As shown in Figure 6, under S3 treatment, the contents of Na, P and S accumulated in the seeds of Huageng5 were significantly higher than those in the seeds of Xudao9 and Yangyugeng2, and the contents of Cu, P, Mg, and K accumulated in the seed of Xudao9 were more than those in Yangyugeng2. The Na/K ratio of Xudao9 was lower than that of Yangyugeng2 and Huageng5, indicating that Xudao9 had the strongest salt tolerance among varieties, while the Na/K ratio of Huageng5 was slightly higher than that of Yangyugeng2. However, the absorption capacity of Huageng5 for other ions was significantly higher than that of Yangyugeng2, and other ions could mitigate the sodium ion poisoning to a certain extent, so the salt tolerance of Huageng5 was significantly stronger than that of Yangyugeng2 (Figure 6).

## Discussion

### Effect of Salinity Stress on Rice Yield and Grain Quality

Rice yield is the result combining yield components that depend on the production capacity of photosynthetic materials and the functioning and distribution of assimilates. Salinity affects rice growth, which in turn affects both rice yield and quality (Turan et al., 2007; Genyou et al., 2018). The salinity stress significantly affects the yield traits of rice, such as seed setting rate, tiller number, panicle number, and paniclelength; among these contributing traits, seed setting rate is the main cause of yield loss under saline conditions (Shereen et al., 2005). However, in this study, we found that the panicle number is considered as the main factor affecting rice yield (Figure 1). A 12% increase in the panicle numbers of Nipponbare was reported when 20 mM NaCl was applied to at 4-weeks after transplanting (Thitisaksakul et al., 2015). In this study, the panicle number increased by 9.68 and 4.78% for Xudao9 and Huageng5, respectively, at a salt concentration of 17.1 mM NaCl, and increased by 4.19% for Xudao9 and decreased by 2.05% for Huageng5 at a salt concentration of 25.6 mM NaCl. In contrast, the panicle number of Yangyugeng2 decreased by 5.76 and 6.92% at these two salt concentrations, respectively (Figure 1A). This difference is closely related to the differential salt tolerance of rice varieties, as well as the timing and methods of salt stress.

Rice quality is particularly important for the health of people who eat it as a staple food (Ali et al., 2020). In comparison with the rate of the non-saline environment, the brown rice rate, milled rice rate and head milled rice rate were significantly declined at 51.3 mM NaCl (Genyou et al., 2018). This study revealed that salt stress could increase brown rice rate, milled rice rate and head milled rate when the salinity level is less than or equal to 34.2 mM NaCl. This indicated that the effect of salinity stress on the contributing quality traits of rice were related to the salinity level of in soils (Figures 2A–C).

Appearance quality of rice determines its capability to attract consumers, and chalkiness is the main indicator of appearance quality (Zhou et al., 2020; Ayaad et al., 2021). Seeds with high chalkiness are easily broken during milling and thus losing marketability (Sawada et al., 2016). Transparent grain with little or no chalkiness, intermediate gel consistency, intermediate amylose content and low starch content are the desired attributes of good quality rice (Rao et al., 2013). When the salt concentration was higher than 34.2 mM NaCl, the quality of milling and appearance of rice were significantly affected, and the protein contents was significantly increased (Chengke et al., 2017). The results described that the chalkiness rate and chalkiness degree of rice seed increased under the salinity stress, except for Yangyugeng2. And the chalkiness rate and degree of Yangyugeng2 declined when the salt concentration was 34.2 mM NaCl (Figures 3A,B). Grain length is more important than width as long grains tend to break during the milling process, and the grain aspect ratio was significantly declined in the salinity soil (Rao et al., 2013). In this study, we demonstrated that the salt stress significantly decreased the length of rice grain but increased the aspect ratio of grain seed except for Huageng5 (Figures 2D,F). It was reported that the application of 20 mM NaCl for 4 weeks after transplanting increased the seed length by 3.01% (Thitisaksakul et al., 2015), but in this study only Yangyugeng2 increased the seed length by 0.4% at a salt concentration of 17.1 mM NaCl (Figure 2D). It was also reported that salt stress (4 ds/m) did not cause any change in seed length, width and aspect ratio compared to the control group (Sangwongchai et al., 2021). It is worth noting that the duration of salt stress in this experiment was only from flowering to maturity. It can be seen that the duration of salt stress and the mode of salt application significantly affected the experimental results.

Amylose content is an important indicator of rice quality, and low-taste rice varieties with high amylose content will expand in volume during cooking, which are less likely to be tender and hard after cooling. Good-taste rice varieties with low amylose content is moist and sticky when being cooked (Juliano, 1971). Protein is the second most important ingredient after starch and the higher protein content means the better quality of rice (Chen et al., 2021). Numerous studies found that the salinity stress lower the nutritional value of rice (Yajuan et al., 2019), such as amylose content and gel consistency (Rao et al., 2013). The amylose content was significantly decreased under salt stress compared with that of no salinity level, while protein content was the opposite (Genyou et al., 2018). It was also reported that there was no significant change in the amylose and protein content of rice seeds under salt stress (4 ds/m) (Sangwongchai et al., 2021). It has further been suggested that high salt stress (EC 6–8 ds/m) increased the seed protein content, while low salt stress (EC 2.5 ds/m) decreased the seed protein content (Sangwongchai et al., 2021). In this study, the protein content of the salt-tolerant varieties showed a decreasing trend followed by an increasing trend, while the salt-sensitive varieties showed a decreasing trend. The amylose content of Xudao9 exhibited an increasing trend followed by a decreasing trend, while Huageng5 showed an increasing trend and Yangyugeng2 did not change significantly (Figures 3C,D). The gel consistency of salt-tolerant varieties tended to decrease and then increase, while that of salt-sensitive varieties tended to increase and then stabilization under salt stress.

The RVA spectrum of starch can better reflect the taste quality of steamed rice, but it is also affected by varieties and environmental conditions (Fan et al., 2021). The RVA curve is a characteristic that can reflect the changes in rice starch viscosity during the rapid heating and cooling of known amounts of rice flour and water (Chen et al., 2021). It has been represented that the RVA value of rice starch was closely related to the taste value of rice, with rice of good quality generally meaning high peak viscosity, high breakdown, and low setback (Genyou et al., 2018). Some scholars concluded that the setback and the pasting temperature increased significantly under 51.3 mM of salt stress, which indicated a negative effect of salt stress on the cooking and taste quality of rice (Genyou et al., 2018). It was also reported that the peak viscosity, hot paste viscosity, cold paste viscosity and breakdown of rice flour reached the maximum value under the salt environment of 17.1 mM NaCl (Dandan et al., 2020). In this study salt stress increased the peak viscosity of Xudao9 and Huageng5, and decreased the setback of Yangyugeng2, increased the setback of Xudao9, without significant change in the setback of Huageng5. The salt stress increased the pasting temperature of Xudao9, but had no significant effect on that of Yangyugeng2 and Huageng5, which difference may be related to the salt concentrations (Figures 5A,E,G).

### Effect of Salinity Stress on the Content of Minerals in Milled Rice

Minerals are important for maintaining human health. But as our body cannot synthesize them, people only obtain them from food (Yuanmei et al., 2016). Numerous studies have disclosed that the distribution pattern of most trace elements in rice kernels is rice bran > husk > brown rice > fine rice (Yuanmei et al., 2016). The saline stress increased the uptake of Na, K, and Mg in rice grains, but the increase in K and Mg was not significant, and the saline stress decreased the uptake of Ca, Fe, Mn, and Zn. The effect of saline stress on the Na content of brown rice and fine rice in Longdao11 was extremely prominent (Yajuan et al., 2019). Under the saline stress, Mg and Fe were significantly positively correlated, and Mg and Na were significantly negatively correlated (Yajuan et al., 2019).

This experiment demonstrated that there was a significant or extremely significant relationship among appearance quality, nutritive quality, cooking/eating quality, and pasting properties. There was no significant difference between mineral content and taste quality, mineral content and pasting properties under salinity stress.

In this study, the sodium ion uptake capacity of Huageng5 was significantly greater than that of Xudao9 and Yangyugeng2. The potassium ion uptake capacity of Huageng5 and Yangyugeng2 was significantly higher than that of Yangyugeng2. It is thus known that the uptake capacity of rice varieties depends on the uptake capacity of sodium ions and potassium ions (Figures 6A,C). In this study, there was a highly significant positive correlation between potassium ion content and disintegration value, and between and protein content, but a highly significant negative correlation between and setback, width and length, and between and straight chain starch content in rice flour. There was a significant negative correlation between the sodium ion content in rice flour and the length of the seeds. The accumulation of mineral content under salt stress affects the nutritional quality of rice, which in turn affects the rice palatability.

In some cases, when the salt concentration in soil was lower than 10.9 mM NaCl, there was no significant difference in rice yield between soils under or without the salinity stress. When the salt concentration was more than 10.9 mM NaCl, the rice yield and grain quality declined sharply with the increase of salt concentration (Weipu, 2014). Thus, the concentration of 10.9 mM NaCl is recommended as a critical salt concentration for saline rice production, and the salt was implemented for 10 days after transplanting (Weipu, 2014). In other cases, salt stress can increase rice yield and improve the quality of rice milling, cooking and nutrition when the salt concentration is less than or equal to 17.1 mM NaCl (Figures 1, 2). Salt stress can decrease rice yield and affect rice quality when the salinity level is more than or equal to 34.2 mM NaCl (Figures 1, 4). It is recommended that 17.1 mM NaCl be used as the critical salt indicator for saline rice production using salt mixing as the adding method, including sodium chloride and sodium sulfate at 2:1 with the soil before transplanting rice (Chengke et al., 2017). However, other researchers suggested 37.6 mM NaCl as a critical salt indicator for rice production in sodic saline soils. In this experiment, sodic saline soil was mixed with natural black soil in proportion to different concentrations of saline soil (Mingxia et al., 2014). In the present study, salinity stress would increase both milling quality and cooking quality of rice, without significant difference in rice yield when the salt concentration was lower than or equal to 17.1 mM NaCl (Figures 1, 2, 4). However, salt stress would significantly decrease rice yield and rice quality when the salinity level was more than 17.1 mM NaCl, which was consistent with the findings of other authors (Chengke et al., 2017). From the above discussion, the reactions of salt stress are linked to the components of salt, adding method of salt, local salt concentration and soil type.

## Conclusion

Salt is a gift from nature and an ancient treasure for storing food. Nowadays, most of the literature elaborate on the harmful effect of salt on crops. But this study finds that the low concentration of salt has an improving effect on the quality of rice. In the future, mudflat saline land can be planted with screened salt-tolerant varieties, provided that a threshold value for these varieties can be determined to control the salt content of saline land within the threshold value, which is conducive to more rational development and utilization of saline land. This study demonstrates that panicle number is the main cause of yield loss under saline conditions, and low concentration of salt stress can increase rice yield, brown rice rate and milled rice rate of rice, and significant increases head milled rice rate of salt-sensitive varieties but decreases in salt-tolerant varieties. The brown rice rate and milled rice rate increased by 0.26–1.04 and 0.01–2.81% under salt stress. Grain length is more sensitive than grain width to salt stress, and the aspect ratio of seeds has significantly correlated with the rice variety, rather than the salt stress. Different varieties of rice exhibit different salt tolerance under salt stress; the three rice varieties in this study, in order of salt tolerance, were Xudao9, Huageng5 and Yangyugeng2. The low concentration of salt stress will increase the appearance, viscosity, degree of balance, and taste value, and decrease the hardness of rice. The differences in starch pasting properties among rice varieties in this study were larger than those caused by salt stress. The defense measures of different rice varieties vary under the salt stress: Xudao9 in a way maintains a low level of Na/K ratio; Huageng5 alleviates sodium poisoning mainly by uptaking other ions. Rice varieties selected with high salt tolerance can be selected for the development and utilization of mudflats, and the low concentration of salt treatment will increase the rice quality. More research can be conducted in the future on the mechanism of increasing rice quality in low salt concentration environment.

## Data Availability Statement

The raw data supporting the conclusions of this article will be made available by the authors, without undue reservation.

## Author Contributions

RZ and QD planned and designed the experiments. RZ, YW, SH, and RL performed and recorded data during the experiments. RZ, SL, and SY statistically analyzed the data and prepared the tables and graphs. RZ wrote the manuscript. YC, HW, QD, and HH approved the final manuscript after review. All authors have read and agreed to the published version of the manuscript.

## Conflict of Interest

HH was employed by Yibang Agriculture Technology Development Co., Ltd. The remaining authors declare that the research was conducted in the absence of any commercial or financial relationships that could be construed as a potential conflict of interest.

## Publisher’s Note

All claims expressed in this article are solely those of the authors and do not necessarily represent those of their affiliated organizations, or those of the publisher, the editors and the reviewers. Any product that may be evaluated in this article, or claim that may be made by its manufacturer, is not guaranteed or endorsed by the publisher.
